# Effect of low-level mechanical vibration on osteogenesis and osseointegration of porous titanium implants in the repair of long bone defects

**DOI:** 10.1038/srep17134

**Published:** 2015-11-25

**Authors:** Da Jing, Shichao Tong, Mingming Zhai, Xiaokang Li, Jing Cai, Yan Wu, Guanghao Shen, Xuhui Zhang, Qiaoling Xu, Zheng Guo, Erping Luo

**Affiliations:** 1 Department of Biomedical Engineering, Fourth Military Medical University, Xi’an, China; 2 Institute of Orthopaedics, Xijing hospital, Fourth Military Medical University, Xi’an, China; 3 Department of Endocrinology, Xijing hospital, Fourth Military Medical University, Xi’an, China; 4 Department of Nursing, Fourth Military Medical University, Xi’an, China

## Abstract

Emerging evidence substantiates the potential of porous titanium alloy (pTi) as an ideal bone-graft substitute because of its excellent biocompatibility and structural properties. However, it remains a major clinical concern for promoting high-efficiency and high-quality osseointegration of pTi, which is beneficial for securing long-term implant stability. Accumulating evidence demonstrates the capacity of low-amplitude whole-body vibration (WBV) in preventing osteopenia, whereas the effects and mechanisms of WBV on osteogenesis and osseointegration of pTi remain unclear. Our present study shows that WBV enhanced cellular attachment and proliferation, and induced well-organized cytoskeleton of primary osteoblasts in pTi. WBV upregulated osteogenesis-associated gene and protein expression in primary osteoblasts, including OCN, Runx2, Wnt3a, Lrp6 and β-catenin. *In vivo* findings demonstrate that 6-week and 12-week WBV stimulated osseointegration, bone ingrowth and bone formation rate of pTi in rabbit femoral bone defects via μCT, histological and histomorphometric analyses. WBV induced higher ALP, OCN, Runx2, BMP2, Wnt3a, Lrp6 and β-catenin, and lower Sost and RANKL/OPG gene expression in rabbit femora. Our findings demonstrate that WBV promotes osteogenesis and osseointegration of pTi via its anabolic effect and potential anti-catabolic activity, and imply the promising potential of WBV for enhancing the repair efficiency and quality of pTi in osseous defects.

Treatment of osseous defects caused by tumor resection, trauma, infection, or osteo-degenerative diseases imposes tremendous socioeconomic burdens. Despite of extensive clinical use of autograft and allograft as bone substitutes, the tissue availability, donor site morbidity or immunological rejection remains to be their major limitations^1^,^2^. Titanium and titanium alloys have exhibited their superiority as load-bearing metal implants because of the excellent biocompatibility and corrosion resistance^3^. However, the mechanical mismatch between titanium implants and surrounding natural bones leads to the stress-shielding effect and accelerates bone resorption, and thus increases the risk of implant loosening^4^. Titanium alloys with porous structures show structural similarity with cancellous bones and also provide favorable pore channels for the transport of nutrients and metabolites^5^,^6^,^7^. However, titanium-based materials are bioinert which are more easily connected with natural bones in the form of mechanical interlock rather than chemical bonding^4^,^7^,^8^. Inadequate osseointegration of titanium alloys remains to be a major clinical limitation, which fails to assure the early fixation and secure long-term stability of implants, especially for osteoporotic patients^9^,^10^. Thus, developing novel techniques to speed up osseointegration of titanium implants holds great clinical significance for increasing the repair efficiency and quality of bone defects.

The skeleton is a highly adaptive system to load bearing which remodels its own structure in response to the external mechanical cues. Exercise or physical activity increases bone quantity and/or quality, whereas lack of skeletal loading results in prominent degradation of bone mass. Substantial evidence over the past decade has revealed that whole-body vibration (WBV) with low intensity (<50 με) and high frequency (20–100 Hz) might become a promising surrogate of physical exercise in the promotion of bone growth. It has been shown that WBV has the capacity of increasing bone mass and quality in both normal and osteoporotic animals induced by ovariectomy, disuse or glucocorticoids^11^,^12^,^13^,^14^,^15^,^16^,^17^. Clinical investigations have substantiated that WBV promoted bone mineral density (BMD) in young and postmenopausal women^18^,^19^,^20^. Studies have also shown the efficiency of the WBV treatment in the promotion of fracture healing of animal long bones^21^,^22^. Due to the low cost, low side effects, simplicity, short duration, and noninvasive nature, WBV represents significant advances over the physical exercise and existing pharmacologically anabolic or anti-catabolic reagents in the preservation of bone density and strength. However, critical questions regarding whether the WBV treatment is able to promote bone ingrowth and accelerate osseointegration of porous metallic implants in animals with bone defect remain unanswered yet.

In the present study, we systematically investigated the potential effects of WBV stimulation on the biological performance of pTi both *in vitro* and *in vivo*. First, osteoblastic activities and functions *in vitro* in pTi under the stimulation of WBV were critically assessed. Then, we evaluated the efficiency of WBV in bone ingrowth through the pore channels and peri-implant osseointegration. Finally, the molecular signaling pathway mechanisms of WBV stimulation on *in vitro* and *in vivo* osteogenesis in pTi implants were also investigated.

## Materials and Methods

### Fabrication of pTi implants

Ti6Al4V pTi structures (70% porosity, 750 μm pore size, 1.33 g/cm^3^ density and 0.3 relative density) were designed with the computer-aided design (CAD) software, and the pTi implants were fabricated using the electron beam melting system (EBM S12, Acram AB, Sweden) as previously described^6^. The Ti6Al4V titanium alloy powder was made into porous cylinders of 2.5 mm in thickness and 12 mm in diameter for *in vitro* experiments and porous discs of 6.0 mm in diameter and 8.0 mm in length for *in vivo* experiments. The prepared pTi implants were scanned with a scanning electron microscope (SEM, JSM-6460, JEOL, Japan) to visualize their microstructure and morphology. All implants were sterilized by autoclaving for 0.5 h prior to experiments.

### *In vitro* and *in vivo* mechanical loading devices

**Figure 1:**
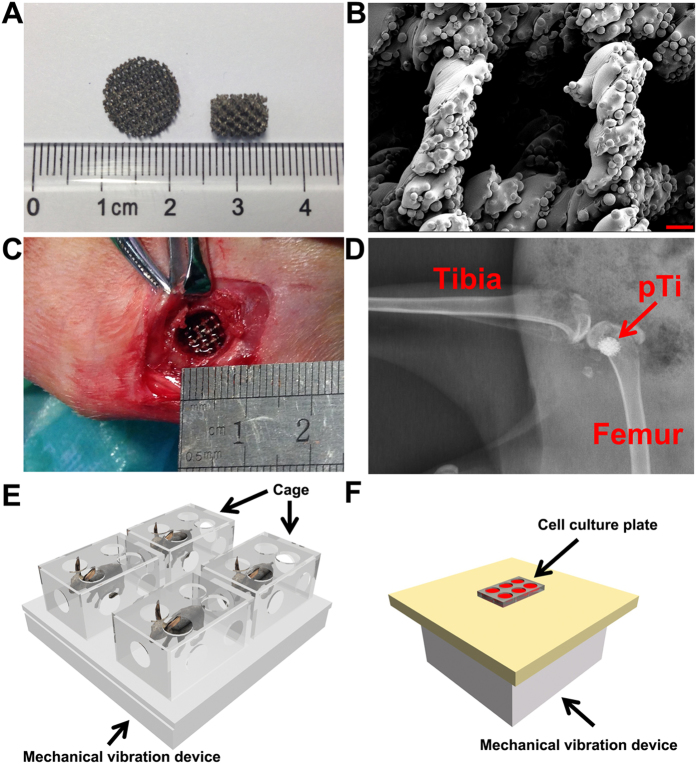
Characterization of Ti6Al4V pTi samples (70% porosity and 750-μm pore size) and WBV system setups for *in vivo* and *in vitro* experiments. **(A)** Gross view of pTi discs used for *in vitro* experiments (12.0-mm diameter and 2.5-mm thickness) and pTi implants used for *in vivo* experiments (6.0-mm diameter and 8.0-mm length). **(B)** Microstructural observation of pTi implants via SEM scanning. Scale bar represents 200 μm. **(C** ~ **D)** Surgical photograph for the establishment of cylindrical bone defect with 6.0-mm diameter and 8.0-mm length in the femoral lateral condyle of rabbits. A pTi implant was then transplanted into the bone defect site and the accuracy of the location of bone defect was further confirmed via X-ray scanning. **(E** ~ **F)** Schematic representation of the WBV systems for *in vivo* and *in vitro* experiments. For *in vivo* WBV stimulation, rabbits were placed onto the WBV platform (100 cm × 100 cm × 30 cm) and confined individually by inverted plastic cages (40 cm × 30 cm × 25 cm). For the *in vitro* experiment, 6-well dishes filled with primary osteoblast-seeded pTi implants were placed on the platform (30 cm × 30 cm × 30 cm). The vertical vibratory motion was generated by an electromagnetic actuator mounted beneath the platform. The two WBV systems generated a sinusoidal waveform with a vertical acceleration of 0.5 g at a frequency of 30 Hz.

Two custom-designed mechanical vibration systems were employed in the present study to generate low-intensity and high-frequency loads to *in vitro* primary osteoblasts and *in vivo* animal skeletons (Fig. 1). For *in vivo* mechanical loading, animals were placed onto the WBV platform (100 cm × 100 cm × 30 cm) and confined individually by inverted plastic cages (40 cm × 30 cm × 25 cm). For *in vitro* mechanical loading, 6-well dishes filled with osteoblast-seeded pTi implants were placed on the platform (30 cm × 30 cm × 30 cm). An electromagnetic actuator controlled by a function generator was mounted beneath the platform to generate the vertical vibratory motion. An accelerometer (VIB-5, Shanghai Xingsheng Detecting Instrument Co., Ltd., China) was closely attached to the WBV platform to determine the mechanical signals transmitted to the cells or animals. The WBV generating systems imposed the vibration loading on both *in vitro* osteoblasts and *in vivo* animal skeletons at a sinusoidal waveform with a vertical acceleration of 0.5 g at a frequency of 30 Hz.

### Cell culture of primary osteoblasts

Primary osteoblasts were obtained by digesting the calvarial bone of 1-day-old New Zealand rabbits according to the previously described protocol^23^. All animal procedures were approved by the Institutional Animal Care and Use Committee of the Fourth Military Medical University, and all procedures were strictly performed in accordance with the approved guidelines. Cells were maintained in α-minimum essential medium (α-MEM; Hyclone, Logan, UT, USA) containing 10% fetal bovine serum (FBS; Hyclone) and 1% penicillin/streptomycin (Hyclone) at 37 °C. Primary rabbit osteoblasts were identified via alkaline phosphatase (ALP) staining and Alizarin Red staining for mineralization nodules^24^. Cells with passages 3 ~ 6 were used in the experiment. Cells were seeded onto the pTi discs at a density of 5 × 10^4^ cells/ml for 12 h. Cells in the WBV group were subjected to 1 h/day mechanical vibratory stimulation for 3 days at room temperature (RT). Cells in the Control group were placed onto the inactivated mechanical loading platform.

### *In vitro* osteoblastic proliferation

3-(4,5-dimethylthiazol-2-yl)-2,5-diphenyltetrazolium bromide (MTT; Sigma) assays were performed to determine osteoblastic proliferation^25^. In brief, after WBV stimulation, the pTi samples seeded with primary osteoblasts were incubated with 80 μL MTT at 37 °C for 4 h. Then, 800 μL dimethyl sulfoxide (DMSO) was added to dissolve the formazan formed by MTT. The mixture was then transferred to 96-well plate and the optical density (OD) values were determined at 490 nm with a multimode microplate reader (Tecan GENios, San Jose, CA, USA).

### *In vitro* osteoblastic mineralization

Osteoblastic mineralization was determined using a quantitative Alizarin red-S staining as previously reported^26^,^27^. In brief, after WBV stimulation for consecutive 7 days (1 h/day), the pTi samples seeded with primary osteoblasts were fixed with 4% paraformaldehyde and then stained with 40 mM Alizarin red-S for 1 h. After rinsed with PBS, the bound stain was eluted with 0.5 ml of 5% cetylpyridinium chloride. The OD values of the solution were determined at 405 nm with a multimode microplate reader (Tecan GENios).

### *In vitro* osteoblastic attachment and morphology examination

After mechanical vibration stimulation, primary osteoblastic cells were fixed in 4% formaldehyde solution for 5 min and permeabilized with 0.1% Triton X-100 to evaluate osteoblastic attachment and morphology. Cells were then stained with 50 mg/ml FITC (Sigma) for 40 min and 40,60-diamidino-2-phenylindole (DAPI; Beyotime Institute of Biotechnology) for 10 min. After washing, cells were visualized and analyzed using a confocal microscope (FV1000, Olympus, Tokyo, Japan) in five randomly selected fields of view. For the SEM examination, the samples after mechanical stimulation were fixed in 1 ml of 2.5% glutaraldehyde solution for 1 h at RT and then dehydrated in a series of acetonitrile washes. All samples were dried to the critical point, coated with gold, and then imaged under the SEM machine (Hitachi JSM-4800).

### *In vitro* osteoblastic osteogenesis-related gene expression

Total RNA was isolated from the implants attached with primary osteoblasts using TRizol (Invitrogen, Carlsbad, CA, USA) and quantified with a spectrophotometry (SmartSpec Plus, Bio-Rad, Hercules, CA, USA). RNA (2 μg) was reverse-transcribed into cDNA in 40 μL system with oligo(dT)_18_ as a primer using FastQuant RT Kit (Tiangen Biotech, Beijing, China). qRT-PCR was performed on 2 μL cDNA in a reaction of 20 μL system with Maxima SYBR Green qPCR (Thermo Fisher Scientific, Waltham, MA, USA) using the Bio-Rad CFX96 real-time PCR detection system (Philadelphia, PA, USA). The primer sequences utilized in qRT-PCR are shown in Table 1. The protocol for qRT-PCR reactions consisted of an initial denaturation at 95 °C for 10 min followed by 40-cycle denaturation at 95 °C for 15 sec, annealing at 55 °C for 15 sec, and extension at 55 °C for 15 sec. β-Actin was used as an internal control for normalization. The relative quantity of mRNA was calculated (2^−△△Ct^ analysis). All qRT-PCR reactions were performed three times.

### *In vitro* osteoblastic osteogenesis-related protein expression

The implants attached with primary osteoblasts were washed with ice-cold PBS and lysed to release the whole proteins by RIPA buffer with 1 mM PMSF. The cell lysates were agitated at 4 °C for 30 min and then centrifuged at 4 °C for 20 min. The protein concentration was determined by the BCA assay. The protein extracts (30 μg per sample) were separated by 8% or 10% Tris-glycine SDS-PAGE and then transferred onto PVDF membranes (Millipore) after mixed with 5× loading buffer. The PVDF membranes were blocked in TBST (Tris Buffer Saline, 0.5% Tween-20) containing 5% BSA for 1 h and incubated overnight at 4 °C with primary antibodies to OCN (1:1000, Abcam, Cambridge, MA, USA), Runx2 (1:1000, Biorbyt Ltd., Cambridge, UK), Wnt3a (1:1000, Novus Biologicals, Littleton, CO, USA), Lrp6 (1:1000, Lifespan Bioscience, Seattle, WA, USA), β-catenin (1:1000, Millipore, Billerica, MA, USA), β-Tubulin (1:3000, Bioworld technology, Inc., Louis Park, MN, USA), and β-Actin (1:3000, Bioworld) in TBST containing 5% BSA. The membranes were then incubated with a 1:3000 dilution of HRP-conjugated secondary antibody for 1 h at RT, and visualized by an ECL chemiluminescence system (GE ImageQuant 350, GE Healthcare). Semi-quantitative analysis was performed using the QuantityOne Software (Bio-Rad). β-Actin or β-Tubulin was used as an internal control for normalization.

### Bone defect animal model

The Institutional Animal Care and Use Committee of the Fourth Military Medical University approved this study, and all procedures were strictly carried out in accordance with the approved guidelines. Twenty-four female New Zealand rabbits (3.0 ± 0.4 kg, Animal Center of the Fourth Military Medical University, Xi’an, China) were acclimatized to the laboratory for 7 days prior to surgery. Animals were anesthetized via intramuscular injection with 3% pentobarbital sodium (30 mg/kg). Left hindlimbs of rabbits were shaved, cleansed with iodophor solution, and covered with sterile drapes. A longitudinal incision in the distal femur was created to expose the lateral condyle. A cylindrical bone defect with 6.0-mm diameter and 8.0-mm length was created with an electrical drill. The defect was then washed with saline and hydrogen peroxide. The drill-hole bone defect in one femur was filled with a matching-size cylindrical block of pTi. The incisions in the muscle, subcutaneous tissue and skin were then sutured, respectively. All surgical procedures were performed aseptically to avoid the potential infection of pathogens. Rabbits received intramuscular injection of penicillin (40000 U) for three consecutive days after surgery. Animals were then randomly and equally assigned to the Control and WBV groups. Rabbits in the WBV group were subjected to 1 h/day WBV stimulation. All animals received intramuscular injections of 8 mg/kg calcein (Sigma-Aldrich, Louis, MO, USA) on 14 and 4 days before sacrifice, respectively. After WBV stimulation for 6 and 12 weeks, 6 rabbits in each group were euthanatized with an overdose of pentobarbital sodium. The femoral condylar samples were immediately harvested and immersed in 80% ethanol for μCT, histological and histomorphometric analyses. The femoral bone with 1-cm height right above the bone defect site was snap-frozen in liquid nitrogen for qRT-PCR analyses.

### μCT analyses

A high-resolution μCT system (Y. Cheetah, YXLON, Germany) was employed to evaluate the microstructure of left femoral condyles of rabbits in each group (*n *= 6 in each time point) with a scanning resolution of 18.2 μm/slice. After scanning, 3-D images were reconstructed based on the acquired 2-D image sequences. A tube volume with 6.0-mm diameter and 8.0-mm length was defined as the volume of interest (VOI), which completely covered the region of the pTi implant. The trabecular bone parameters, including bone volume per tissue volume (BV/TV), bone surface per bone volume (BS/BV), trabecular number (Tb.N), trabecular thickness (Tb.Th) and trabecular separation (Tb.Sp) were quantified.

### Histology and histomorphometry

After μCT scanning, a diamond saw microtome (Leica 2500E, Leica SpA, Milan, Italy) was used to section the pTi samples longitudinally along the pTi implants (~50 μm thick). The calcein double-labeling sections were imaged with a fluorescence microscope (LEICA DM LA, Leica Microsystems, Heidelberg, Germany) to quantify the dynamic histomorphometric parameters, including mineral apposition rate (MAR), mineralizing surface per bone surface (MS/BS) and bone formation rate per bone surface (BFR/BS). After calcein double-labeling imaging, all samples were subjected to Masson-Goldner trichrome staining for further evaluating the cancellous bone histology inside pTi. The parameter of bone area fraction was quantified from the pixels representing bone tissue (bone area per total area) in the Masson-Goldner trichrome staining images.

### *In vivo* osteogenesis-related gene expression

Before RNA extraction, samples were immediately crushed into powder in a mortar containing liquid nitrogen using the pestle and then mixed with the monophasic solution of phenol and guanidine thiocyanate. Total RNA was extracted using the guanidinium isothiocyanate-alcohol phenyl-chloroform method. Then, the FastQuant RT Kit was used to synthesize cDNA from RNA. qRT-PCR was performed on the Bio-Rad CFX96 real-time PCR system. The primer sequences utilized in qRT-PCR are shown in Table 1. All mRNA levels were normalized by the house-keeping gene β-Actin. The relative quantity of mRNA was calculated (2^–△△Ct^ analysis). All qRT-PCR reactions were performed three times.

### Statistical analysis

All data presented in this study were expressed as the mean ± standard deviation (S.D.). Statistical analyses were performed using SPSS version 13.0 for Windows software (SPSS, Chicago, IL, USA). The differences of each parameter between the Control group and WBV group were examined using a Student t-test. The significance level was set at 0.05.

## Results

### *In vitro* osteoblastic attachment, proliferation, mineralization and morphology in pTi

**Figure 2:**
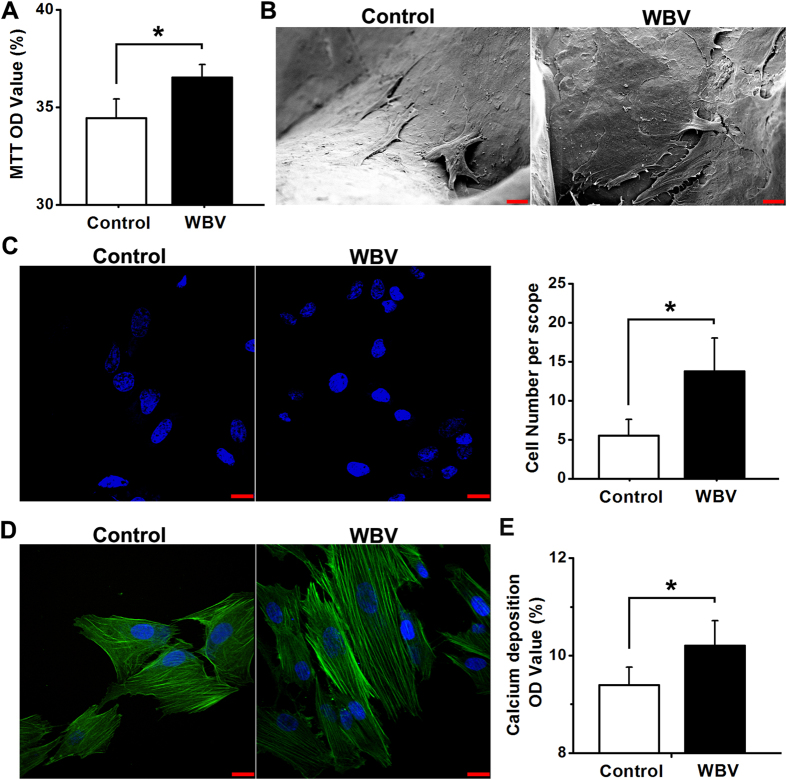
Effects of mechanical vibration stimulation on *in vitro* cellular attachment, proliferation, mineralization and morphology for primary rabbit osteoblasts seeded in pTi. **(A)** Comparisons of *in vitro* osteoblastic proliferation between the Control and WBV groups via MTT assays (*n *= 5). **(B)** Representative SEM scanning for *in vitro* primary osteoblasts seeded in pTi in the Control and WBV groups. Scale bar represents 10 μm. **(C)** Comparisons of *in vitro* osteoblastic attachment between the Control and WBV groups via DAPI staining (*n *= 4). Scale bar represents 20 μm. **(D)** Representative *in vitro* osteoblastic FITC cytoskeleton staining images in the Control and WBV groups. Scale bar represents 20 μm. **(E)** Comparisons of *in vitro* osteoblastic mineralization between the Control and WBV groups via quantitative Alizarin red-S staining (*n *= 15). Values are all expressed as mean ± S.D. ^*^Significant difference from the Control group with *P* < 0.05.

Primary rabbit osteoblasts exhibited the fusiform, triangle or polygonal shape with round or oval nucleus, and also demonstrated positive in ALP and mineralization nodules staining (data not shown). As shown in Fig. 2A, mechanical vibration stimulation significantly promoted osteoblastic proliferation via MTT analysis (*P* < 0.05). Images by SEM scanning showed that primary osteoblasts were attached to the substrate of titanium alloy more tightly and proliferated with many more pseudopodia in pTi under the stimulation of mechanical vibration (Fig. 2B). Moreover, mechanical vibration stimulation significantly increased osteoblastic attachment in pTi as compared with the Control group via DAPI staining (Fig. 2C, *P* < 0.05). FITC cytoskeleton staining images (Fig. 2D) show that osteoblasts in the WBV group displayed well-developed cytoskeleton with higher fluorescence intensity, more microfilaments with directional arrangement, and thicker stress fibers than the cells in the Control group. As shown in Fig. 2E, mechanical vibration induced significant increase of the formation of mineralized nodules for primary osteoblasts seeded in pTi (*P* < 0.05).

### *In vitro* osteogenesis-associated gene and protein expression

**Figure 3:**
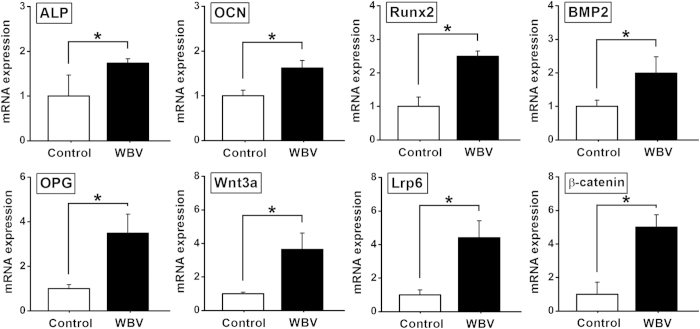
Effects of mechanical vibration stimulation on *in vitro* osteogenesis-related gene expression for primary rabbit osteoblasts seeded in pTi via qRT-PCR analyses, including ALP, OCN, Runx2, BMP2, OPG, Wnt3a, Lrp6 and β-catenin. Values are all expressed as mean ± S.D. (*n *= 4), and the relative expression level of each gene was normalized to β-Actin. ^*^Significant difference from the Control group with *P* < 0.05.

**Figure 4:**
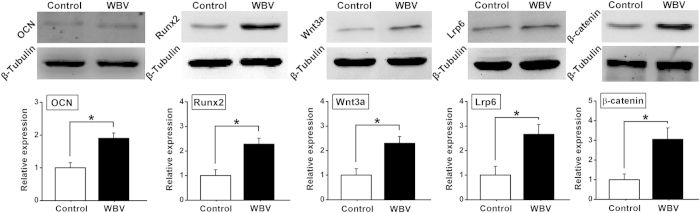
Effects of mechanical vibration stimulation on *in vitro* osteogenesis-related protein expression for primary rabbit osteoblasts seeded in pTi via western blotting analyses, including OCN, Runx2, Wnt3a, Lrp6 and β-catenin. Values are all expressed as mean ± S.D. (*n *= 3 ~ 4). The relative expression level of each protein was normalized to β-Tubulin. ^*^Significant difference from the Control group with *P* < 0.05.

As shown in Fig. 3, mechanical vibration significantly promoted ALP, OCN, Runx2, BMP2, OPG gene expression as compared with the Control group (*P* < 0.05). The gene expression levels of the canonical Wnt signaling pathway (including Wnt3a, Lrp6 and β-catenin) were significantly higher in the WBV group than those in the Control group (*P* < 0.05). Moreover, western blotting results (Fig. 4) reveal that mechanical vibration stimulation significantly stimulated OCN, Runx2, and canonical Wnt signaling (including Wnt3a, Lrp6 and β-catenin) protein expression as compared with the Control group (*P* < 0.05).

### μCT analysis for *in vivo* osseointegration of pTi

**Figure 5:**
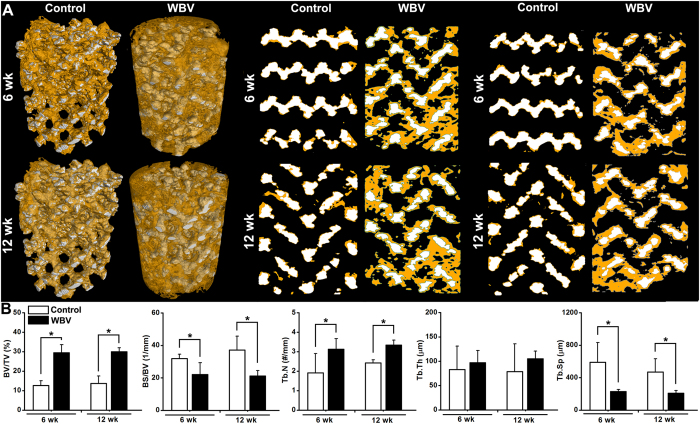
Effects of WBV stimulation for 6 weeks and 12 weeks on the osteogenesis and osseointegration of pTi implants in the region of bone defects via μCT scanning. A tube volume with 6.0-mm diameter and 8.0-mm length was defined as the volume of interest (VOI), which completely covered the region of the pTi implant. (**A**) Reconstructed 3-D μCT images determined by the VOI and 2-D mid-coronal and mid-sagittal slices. The regions with white color represent titanium alloys and the areas with yellow color represent cancellous bones. **(B)** Quantitative comparisons of μCT characteristic parameters of trabecular bones between the Control and WBV groups (*n* = 6), including bone volume per tissue volume (BV/TV), bone surface per bone volume (BS/BV), trabecular number (Tb.N), trabecular thickness (Tb.Th) and trabecular separation (Tb.Sp). Values are all expressed as mean ± S.D. ^*^Significant difference from the Control group with *P* < 0.05.

Representative 3-D μCT images (Fig. 5A) demonstrate that the amount of newly formed bone within the pTi implant was significantly increased after 6-week or 12-week WBV stimulation in comparison with the Control group. Representative 2-D mid-coronal and mid-sagittal slices further reveal that WBV stimulation for 6 weeks and 12 weeks significantly promoted bone ingrowth through the pores of pTi. Moreover, WBV stimulation resulted in higher levels of BV/TV (*P* < 0.05, +133.5% at 6 weeks and +118.1% at 12 weeks) and Tb.N (*P* < 0.05, +63.4% at 6 weeks and +37.5% at 12 weeks) as revealed by statistical comparisons (Fig. 5B). WBV also significantly decreased BS/BV (*P* < 0.05, −30.8% at 6 weeks and −43.0% at 12 weeks) and Tb.Sp (*P* < 0.05, −61.3% at 6 weeks and −55.7% at 12 weeks) as compared with the Control group. In addition, WBV exposure also resulted in minor increase of Tb.Th at 6 weeks (*P* = 0.536, +17.2%) and 12 weeks (*P* = 0.219, +33.2%).

### Histological and histomorphometric evaluation for *in vivo* osseointegration of pTi

**Figure 6:**
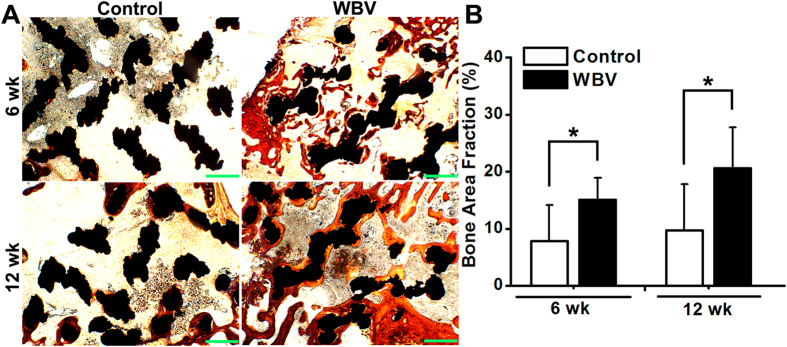
Effects of WBV stimulation for 6 weeks and 12 weeks on cancellous bone histology in the region of bone defects via Masson-Goldner trichrome staining. (**A**) Representative histological images for bone microarchitecture in the region of bone defects by Masson-Goldner trichrome staining. The black areas represent titanium alloys and the red areas represent cancellous bones. Scale bar equals 100 μm. (**B**) Quantitative comparisons of bone area fraction (bone area per total area) determined by the histological analyses between the Control and WBV groups (*n* = 6). Values are all expressed as mean ± S.D. ^*^Significant difference from the Control group with *P* < 0.05.

**Figure 7:**
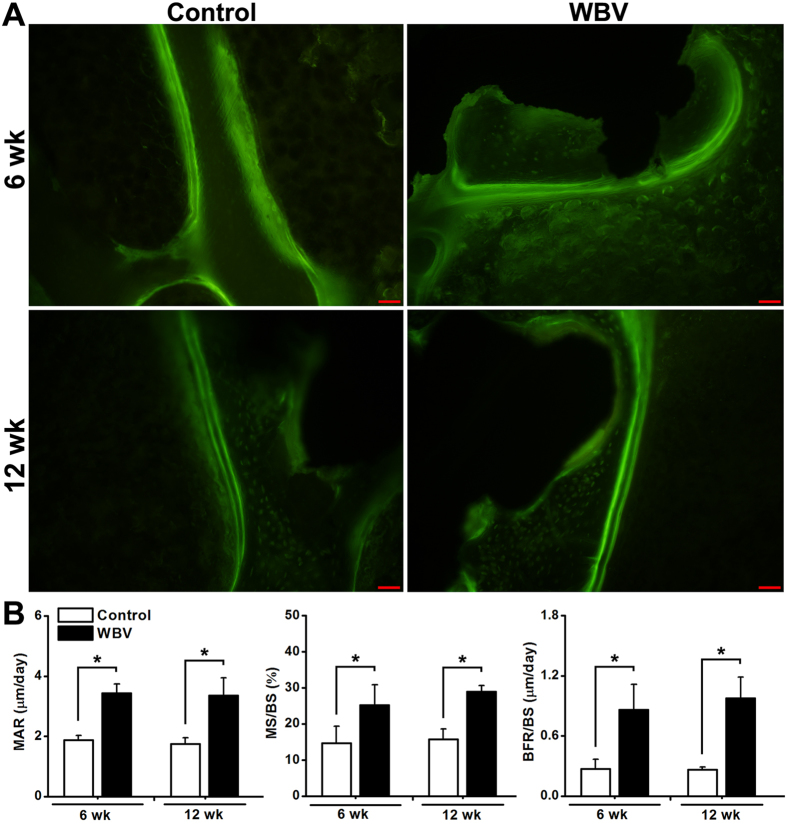
Effects of WBV stimulation for 6 weeks and 12 weeks on dynamic histomorphometric parameters in the region of bone defects via double calcein labeling. (**A**) Representative calcein double-labeling sections in the region of bone defects. Scale bar represents 100 μm. (**B**) Quantitative comparisons of the dynamic histomorphometric parameters, including mineral apposition rate (MAR), mineralizing surface per bone surface (MS/BS) and bone formation rate per bone surface (BFR/BS) between the Control and WBV groups (*n *= 6). Values are all expressed as mean ± S.D. ^*^Significant difference from the Control group with *P* < 0.05.

As shown in Fig. 6A, Masson-Goldner trichrome staining images revealed that 6-week and 12-week WBV stimulation stimulated more new trabecular bone ingrowth through the pores of pTi in the region of bone defects. The findings were further confirmed by the statistically significant increase of bone area fractions after 6-week and 12-week WBV exposure as compared with the Control group (Fig. 6B, *P* < 0.05, +92.2% at 6 weeks and +112.1% at 12 weeks). Effects of WBV stimulation on dynamic histomorphometric parameters in the region of bone defects via calcein double-labeling analyses are shown in Fig. 7A. WBV stimulation for 6 weeks and 12 weeks remarkably speeded up the new bone formation in the region of bone defects. Furthermore, quantitative comparisons of the dynamic histomorphometric parameters (Fig. 7B) demonstrate that WBV exposure significantly promoted the levels of MAR (*P* < 0.05, +82.7% at 6 weeks and +91.9% at 12 weeks), MS/BS (*P* < 0.05, +72.3% at 6 weeks and +84.3% at 12 weeks) and BFR/BS (*P* < 0.05, +216.7% at 6 weeks and +268.3% at 12 weeks) as compared with the Control group.

### *In vivo* osteogenesis-associated gene expression

**Figure 8:**
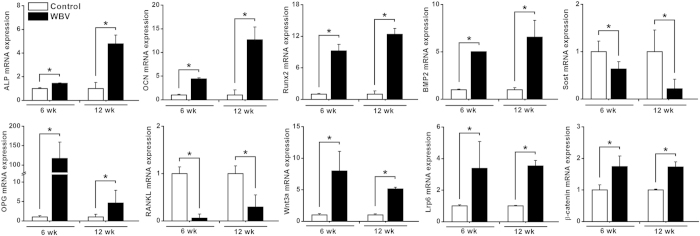
Effects of WBV stimulation for 6 weeks and 12 weeks on *in vivo* osteogenesis-related gene expression in rabbit femora via qRT-PCR analyses, including ALP, OCN, Runx2, BMP2, Sost, OPG, RANKL, Wnt3a, Lrp6 and β-catenin. Values are all expressed as mean ± S.D. (*n* = 3 ~ 6) and the relative expression level of each gene was normalized to β-Actin. ^*^Significant difference from the Control group with *P* < 0.05.

Effects of WBV stimulation on *in vivo* osteogenesis-related gene expression levels in rabbit femora via qRT-PCR analyses are shown in Fig. 8. WBV stimulation for 6 weeks and 12 weeks resulted in significant increases of ALP, OCN, Runx2, BMP2 and OPG (*P* < 0.05), and also induced significant decreases of Sost and RANKL (*P* < 0.05). Furthermore, the mRNA expression levels of the canonical Wnt signaling, including Wnt3a, Lrp6 and β-catenin in the WBV group were also significantly higher than those in the Control group (*P* < 0.05).

## Discussion

Titanium alloys with porous structure have the capacity of providing favorable environment for the support of tissue adhesion and vascularization, as well as minimizing the stress-shielding issues^5^,^28^,^29^. However, exploring effective methods for the enhancement of osseointegration and more adequate ingrowth of mineralized bone tissue into the pore spaces of pTi remains a major clinical concern, which is helpful for providing stable long-term anchorage for biological fixation of implants. In the present study, WBV, as a kind of safe, affordable and non-invasive biophysical intervention, was used to determine its potential for improving the osteogenesis and osseointegration of pTi implants. Firstly, our *in vitro* findings show that mechanical vibration significantly promoted osteoblastic activities and enhanced osteogenesis in pTi. Secondly, the *in vivo* experiments demonstrate that WBV was able to stimulate osteogenesis and osseointegration, and enhance bone formation in pTi implants. Moreover, we also reveal that WBV regulated peri-implant bone remodeling by mediating a series of molecular signaling associated with osteoblastogenesis and osteoclastogenesis, including BMP2, Wnt/Lrp6/β-catenin, Sost and OPG/RANKL/RANK. The present study demonstrates that WBV might become a potential biophysical modality for enhancing the repair efficiency and quality of pTi in long bone defects.

Cell adhesion onto biomaterials is considered to be the major prerequisites for osteoblasts exerting their biological functions^30^, which involves a complex process with a series of chemical responses, including serum-protein adsorption, cell contact, attachment, and spreading^31^,^32^. Our *in vitro* results show that micromechanical vibration with 0.5 g, 30 Hz promoted more osteoblasts adhered onto the pTi substrate. Furthermore, the SEM scanning results revealed stronger interlocking of cells into the titanium surface after mechanical vibration stimulation. Thus, the enhancement of cell adhesion by mechanical vibration played a critical role in the initiation of the activation of subsequent intracellular biochemical events. The FITC fluorescence staining results further show that osteoblastic cytoskeleton exhibited more and thicker microfilaments with directional arrangement after the stimulation of mechanical vibration. Sato *et al.* have also reported the similar findings in vibration-induced cytomorphological changes of osteoblasts on titanium with smooth surface^33^. These changes in cellular structures are considered to be essential events for bone cells detecting and transducing the external mechanical signals, and thus regulating the biological behavior of bone cells (*e.g.*, proliferation and differentiation)^34^,^35^. The present study also reveals that mechanical vibration stimulated the proliferation and mineralization of primary osteoblasts inside the 3D pTi disks via the MTT analysis and quantitative Alizarin red-S staining. Several previous studies have also demonstrated that vibration loading with 30 Hz significantly increased the cell density of *in vitro* osteoblasts and mesenchymal stromal cells on culture-grade polystyrene dishes^33^,^36^. Together, our findings suggest that mechanical vibration was able to promote cell adhesion, and thus regulate the subsequent biological functions of *in vitro* osteoblasts in 3D pTi implants.

To explore the mechanism by which mechanical vibration regulated osteoblastic activities in pTi, gene and protein expression of osteogenesis-associated molecules and signaling pathways was investigated. Our results show that the gene expression of ALP, an indicator of osteoblast phenotype, was upregulated after mechanical vibration in pTi, which keeps consistent with the previous findings on monolayer cells^37^ or cells seeded on flat titanium surface^33^. We also found that vibration loading enhanced the gene and protein expression of OCN, which is a major osteoblastic differentiation marker. The similar findings were also revealed by Tanaka *et al.* in monolayer MC3T3-E1 cells^37^. Mechanical vibration also resulted in the upregulation of gene and protein levels of Runx2, a key transcription factor involved in osteoblast differentiation^38^. The gene levels of BMP2 and OPG, two key molecules responsible for promoting osteoblast differentiation and inhibiting osteoclast activities, respectively^39^,^40^, were elevated after mechanical vibration stimulation. Moreover, our study reveals the activation of canonical Wnt signaling after the stimulation of mechanical vibration, as evidenced by increased gene and protein expression of Wnt3a, Lrp6 and β-catenin. Our results keep consistent with previous findings, which demonstrate significant upregulation of Wnt3a in bone cells under fluid shear stimulation^41^,^42^. Wnts are a family of secreted proteins existing extensively within the skeleton. Extracellular Wnts can bind to the Frizzled and LRP5/6 co-receptors on the cell membrane, and thus lead to the stabilization of β-catenin in the cytoplasm. Substantial evidence has revealed that activation of canonical Wnt signaling can promote osteoblastogenesis and osteoblast/osteocyte activity^43^,^44^. Numerous studies have also proved that Lrp6 positively regulates osteoblastic activity and bone homeostasis, and mutant mice lacking Lrp6 exhibited decreased BMD^45^,^46^. It is shown that canonical Wnt signaling is also able to activate ALP, Runx2 and BMP2 expression^47^,^48^,^49^. Thus, our findings indicate that canonical Wnt signaling has been implicated in regulating *in vitro* osteoblastic activities in pTi implants.

It has been reported that the optimal pore size of an ideal porous implant biomaterial for the repair of osseous defects should not be lower than 150 μm^50^,^51^. The porosity should exceed 20%, and a higher porosity is able to ensure more adequate *in vivo* tissue response to implantation^5^,^52^. The structural parameters of pTi in the present study, including the porosity and pore size have been widely used in previous investigations both experimentally and clinically^6^,^52^,^53^. Our μCT results exhibited notable enhancement of osteogenesis at the implant-bone interface as well as in the center of the porous implants after 6-week and 12-week WBV stimulation, as evidenced by increased BV/TV, Tb.N, and decreased Tb.Sp and BS/BV. These findings were further confirmed by the histological analysis via Masson-Goldner trichrome staining. Thus, our findings demonstrate that WBV stimulation is able to result in faster and higher-quality osseointegration, which is beneficial for long-term stability and durability of implant fixation, as well as better overall mechanical performance of whole bone^54^,^55^. Unlike the vigorous weight-bearing activities (*e.g.* aerobics and resistance exercises) with more prominent bone stress, micromotion induced by WBV has the capacity of minimizing the discomfort and potential risk of implant movement or loosening. Moreover, dynamic histomorphometric analysis with double calcein labeling showed that WBV speeded up peri-implant bone mineral deposits and increased peri-implant bone formation rate. These results keep in line with previous findings for the regulatory role of WBV in bone remodeling in normal and osteoporotic animals^14^,^17^,^56^. Thus, it is shown in the present study both histologically and ultrastructurally that WBV is able to promote osteogenesis and osseointegration of pTi in the repair of long bone defect by regulating peri-implant bone remodeling.

Then, we investigated the potential molecular signaling mechanisms of WBV on *in vivo* osteogenesis in pTi implants. In line with our *in vitro* findings, WBV resulted in prominent upregulation of ALP, OCN, Runx2 and BMP2, revealing the promotive effects of WBV on osteoblastogenesis and mineralization in peri-implant bones. Furthermore, our results demonstrate that WBV enhanced gene expression in canonical Wnt signaling, as evidenced by increased Wnt3a, Lrp6 and β-catenin mRNA levels. Thus, coupled with the *in vivo* histological and histomorphometric findings together with the *in vitro* results, the present study indicates that WBV promoted skeletal anabolic activities through a canonical Wnt signaling-associated mechanism. Sclerostin, the product of the Sost gene, is exclusively secreted by osteocytes which are regarded as the major mechanosensors in bone^57^,^58^. Sclerostin has proven to be an osteocyte-expressed negative regulator of bone formation, and decreased mechanical loads to bones resulted in significant increases of sclerostin^59^,^60^. Our results show that Sost gene expression was significantly lower in the WBV-stimulated skeletons, which is consistent with the results of Yadav *et al.* who found decreases of Sost expression in the mouse molar after the WBV stimulation^61^. Thus, WBV-induced reduction of Sost expression is also able to facilitate the increase of bone formation rate in pTi implants. Moreover, our results also show increased OPG and decreased RANKL gene expression. It has been proved that the RANKL/OPG ratio is a major predictor of increased osteoclast activity and bone resorption^40^,^62^. Therefore, our results reveal the regulatory role of WBV in peri-implant bone remodeling with potent anabolic effects and potential anti-catabolic effects.

In conclusion, this study opens a new avenue for improving the repairing efficiency and quality of pTi in bone defects in an easy and non-invasive manner. We show that mechanical vibration promoted *in vitro* osteoblastic activities and osteogenesis through a mechanism associated with the activation of canonical Wnt signaling. Our *in vivo* findings demonstrate that WBV promoted osteogenesis and osseointegration of pTi via obvious anabolic actions in the repair of long bone defects. Our *in vivo* molecular signaling investigation further reveals that WBV activated osteoblastogenesis-associated canonical Wnt signaling and also inhibited osteoclastogenesis-associated RANKL/OPG ratio. Thus, our findings suggest that pTi implants accompanied by WBV stimulation exhibit high efficiency and quality in the repair of long bone defects, and might eventually become a clinically applicable treatment modality for osseous defects.

## Additional Information

**How to cite this article**: Jing, D. *et al.* Effect of low-level mechanical vibration on osteogenesis and osseointegration of porous titanium implants in the repair of long bone defects. *Sci. Rep.* **5**, 17134; doi: 10.1038/srep17134 (2015).

## Figures and Tables

**Table 1:** The sequence of primers used in the present study for *in vitro* and *in vivo* real-time fluorescence quantitative PCR.

Genes	Primers	Primer Sequence (5′-3′)	Product Length (bp)
ALP	Forward	ACGGGGCGTGTATCCTCCAA	182
	Reverse	CCCAAGGAGGCAGGATTGAC	
OCN	Forward	TTGGTGCACACCTAGCAGAC	187
	Reverse	ACCTTATTGCCCTCCTGCTT	
Runx-2	Forward	CAGTCTTACCCCTCTTACC	130
	Reverse	CATCTTTACCTGAAATGCG	
BMP2	Forward	GGACGACATCCTGAGCGAGT	117
	Reverse	CGGCGGTACAAGTCCAGCAT	
Sost	Forward	TCTCCCTAGCCCTGTGTCTCCT	100
	Reverse	ACTTCCGTGGCGTCATTCTTGA	
Wnt3a	Forward	ATGAACCGCCACAACAAC	190
	Reverse	GCTTCTCCACCACCATCT	
Lrp6	Forward	GCTTGGCACTTGTATGTAAA	179
	Reverse	TGGGCTAAGATCATCAGACT	
β-catenin	Forward	GACACGGACCACACGCACAA	173
	Reverse	CCGAGCAGCAGCAAGTCTTCT	
OPG	Forward	AACGGCGGCATAGTTCACAAGA	170
	Reverse	GCTGCGAAGCTGATCCAAGGT	
BMP2	Forward	GGACGACATCCTGAGCGAGT	117
	Reverse	CGGCGGTACAAGTCCAGCAT	
β-Actin	Forward	TACGCCAACACGGTGCTGTC	187
	Reverse	ACATCTGCTGGAAGGTGGAGAG	
